# Use acupuncture to relieve perimenopausal syndrome: study protocol of a randomized controlled trial

**DOI:** 10.1186/1745-6215-15-198

**Published:** 2014-05-30

**Authors:** Ying Li, Hui Zheng, Qianhua Zheng, Ling Zhao, Erqi Qin, Yu Wang, Qian Zeng, Huabin Zheng, Yu Zhao, Wei Sun, Xiaoxia Zhang, Zhishun Liu, Baoyan Liu

**Affiliations:** 1 Chengdu University of Traditional Chinese Medicine, 37 Shi’er Qiao Road, Jinniu District, Chengdu, Sichuan 610075, PR China; 2 General Hospital of Chengdu Military Region of the Chinese People’s Liberation Army, 270 Tianhui Road, Rongdu Avenue, Chengdu, Sichuan 610083, PR China; 3 First Affiliated Hospital of Chengdu University of Traditional Chinese Medicine, 39 Shi’er Qiao Road, Jinniu District, Chengdu, Sichuan 610072, PR China; 4 Chengdu Integrated TCM & Western Medicine Hospital, 18 North Wanxiang Road, Gaoxin District, Chengdu, Sichuan 610016, PR China; 5 Guang’anmen Hospital, China Academy of Chinese Medical Sciences, No. 5 BeiXianGe Street, XiCheng District, Beijing 100053, PR China

**Keywords:** acupuncture, randomized controlled trial, perimenopausal syndrome, study protocol

## Abstract

**Background:**

Whether acupuncture is effective for relieving perimenopausal syndrome has been controversial recently. In this article, we report the protocol of a randomized controlled trial using acupuncture to treat perimenopausal syndrome, aiming to answer this controversy.

**Design:**

A multicenter randomized controlled trial with two parallel arms is underway in China. Two hundred and six women with perimenopausal syndrome will be randomly assigned to a treatment group using acupuncture plus auricular acupressure (AA group) and a control group using Climen® (Bayer Healthcare Company Limited, Guangzhou, China), a 28-day sequential hormone replacement therapy, in a 1:1 ratio. Participants in the AA group will receive three acupuncture sessions per week in the first 4 weeks and two sessions per week in the following 8 weeks, for a total of 28 sessions over 12 weeks. Auricular points will be plastered by Semen Vaccariae twice per week for a consecutive 12 weeks, with both ears used alternately. The Climen® control group is prescribed a tablet containing estradiol valerate 2 mg/day for the first 11 days, and a tablet containing estradiol valerate 2 mg/day plus cyroterone acetate 1 mg/day for the following 10 days. The total treatment period of the control group is three cycles. The post-treatment follow-up period will last 24 weeks. The primary outcome is the Menopause Rating Scale (MRS) assessed at baseline and 4, 12, 16, 24 and 36 weeks after randomization. The secondary outcomes are Menopause-Specific Quality of Life, average hot flash score during 24 hours, serum estradiol, follicle-stimulating hormone and luteinizing hormone level. The first two secondary outcomes are measured at the same point as the MRS. Other secondary outcomes are measured at baseline and 12, 24 weeks after randomization.

**Discussion:**

The results of this trial, which will be available in 2015, will clarify whether acupuncture is effective to relieve perimenopausal syndrome.

**Trial registration:**

ClinicalTrials.gov NCT01933204 (registered 9 August 2013)

## Background

Perimenopause is defined as a period during 2 to 8 years preceding menopause and 1 year after final menstruation [1]. During this period, the changes in serum hormone levels – especially estrogen – cause perimenopausal syndrome, manifested as menstrual irregularities, hot flashes, sweats, insomnia, emotional disorders, vaginal dryness, sexual dysfunction, and so forth. The syndrome significantly reduces the women’s health-related quality of life [2,3]. Hormone replacement therapy (HRT) is an effective method to relieve these symptoms [4]. According to recent studies, however, HRT may increase the risk of coronary heart disease [5], stroke [6], breast cancer [7], and endometrial cancer [8]. The benefits of estrogen plus progestin are unable to counterbalance the risks, indicated by the Women’s Health Initiative [9]. More and more women therefore seek alternative therapies rather than HRT, because of the life-threatening side effects [10,11].

As one of the complementary and alternative therapies without serious side effects [12,13], acupuncture is used frequently to treat a wide range of health problems [14], including hot flashes, which is one of the most common symptoms and the reason why women in the stage of menopausal transition seek medical care [15]. However, several randomized controlled trials (RCTs) indicated that acupuncture is not superior to sham acupuncture in relieving perimenopausal symptoms [16-19]. Reliable and valid evidence for the effectiveness of treating perimenopausal syndrome with acupuncture is still inadequate, due to the number, size, design scheme, and quality of recent studies. We therefore designed a multicenter RCT to address these problems, anticipating providing a more conclusive answer to these questions.

In this trial we primarily aim at investigating whether acupuncture is effective and safe in treating perimenopausal syndrome through a comparison with HRT. This work reported in this article is financed by the National Key Technology R&D Program during the Twelfth Five-year Plan Period of China, and is registered with identifier NCT01933204 by ClinicalTrials.gov in the USA.

## Method and design

### Study design

**Figure 1:**
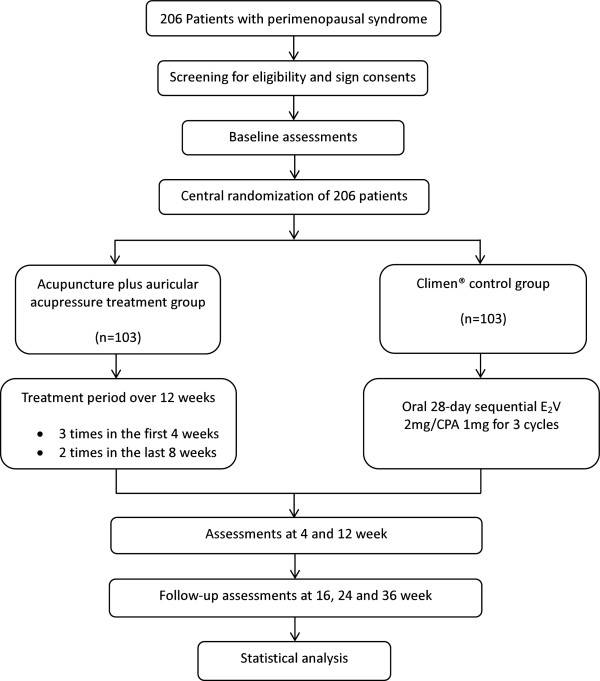
**Trial flow chart.** CPA, cyroterone acetate; E_2_V, estradiol valerate. Climen® from Bayer Healthcare Company Limited (Guangzhou, China).

This study is a multicenter RCT comparing an acupuncture plus auricular acupressure (AA) treatment group with a Climen® (Bayer Healthcare Company Limited, Guangzhou, China) control group (Figure 1, Table 1). Two hundred and six participants will be included from the following three hospitals in Chengdu, Sichuan, China: First Affiliated Hospital of Chengdu University of Traditional Chinese Medicine (TCM), Chengdu Integrated TCM & Western Medicine Hospital, and General Hospital of Chengdu Military Region of the Chinese People’s Liberation Army.

**Table 1:** Trial processes chart

	**Baseline**	**Treatment phase**			**Follow-up phase**		
	**Week −1**	**Week 0**	**Week 4**	**Week 12**	**Week 16**	**Week 24**	**Week 36**
Patients							
Enrollment	×						
Informed consent	×						
Signed informed consent		×					
Medical history	×						
Physical examination	×		×	×			
Laboratory test	×			×		×	
Breast and gynecological ultrasound	×			×			
Randomization		×					
Expectation of acupuncture		×					
Intervention							
Acupuncture plus auricular acupressure treatment group (*n* = 103)			28 sessions of acupuncture				
Comparison							
Climen® control group (*n* = 103)			E_2_V 2 mg/day for days 1 to 11 and E_2_V 2 mg + CPA 1 mg/day for days 12 to 21				
Outcomes							
MRS		×	×	×	×	×	×
MENQOL		×	×	×	×	×	×
Average hot flash score during 24 hours		×	×	×	×	×	×
Serum estradiol, FSH and LH levels	×			×		×	
Patients’ satisfaction			×	×			
Trial evaluation							
Safety of acupuncture			×	×			
Cost–benefit analysis				×			
Reasons of drop-outs or withdrawals							×
Patients’ compliance							×
Adverse events							×
Medicine alliance							×

CPA, cyroterone acetate; E_2_V, estradiol valerate; FSH, follicle-stimulating hormone; LH, luteinizing hormone; MRS, Menopause Rating Scale; MENQOL, Menopause-Specific Quality of Life. Climen® from Bayer Healthcare Company Limited (Guangzhou, China).

These participants will be randomly assigned to two groups (AA treatment group and Climen® control group) through central randomization in a 1:1 ratio. The central randomization system will be used and performed by the Clinical Evaluation Center of China Academy of Chinese Medical Sciences in Beijing. Authorized researchers can obtain random numbers and group assignments by calling the central randomization service number or logging into the website to apply. This procedure assures that randomization will not be influenced either by the acupuncturist or the patients.

The total observation period within this study is 37 weeks, including 1 week of baseline, 12 weeks of treatment and 24 weeks of follow-up. After randomization, participants will receive 28 sessions of acupuncture over a period of 12 weeks plus auricular acupressure or take Climen® (from Bayer Healthcare Company Limited, Guangzhou, China) tablets for three cycles. Acupuncture will be given as three sessions weekly in the first 4 weeks and two sessions weekly in the following 8 weeks for each patient. Each session will last 30 minutes. The follow-up period will last 24 weeks (13 to 36 weeks after randomization). Patients will be assessed before randomization as well as 4, 12, 16, 24 and 36 weeks after randomization.

### Ethics

This study was approved by Sichuan Regional Ethics Review of Committee on Traditional Chinese Medicine in June 2013 (No. 2013KL-013), and follows the principles of the Declaration of Helsinki (Version Edinburgh 2000). All participates are asked to sign the written informed consent before randomization. All patients will be informed and given enough time to decide whether they would sign the informed consent and participate in this trial or they will be given other treatment options if they are not willing to.

### Patients

#### Study population and sample size

This study focuses on women experiencing perimenopausal syndrome. According to the previous study by Guang’anmen Hospital, China Academy of Chinese Medical Sciences (results still unpublished), the improvement of Menopause Rating Scale (MRS) scores of the electro-acupuncture group and the sham acupuncture group were 6.5 and 2, respectively. The difference in scores between the two groups was 4.5, and electro-acupuncture was not superior to sham acupuncture. Accordingly, we assume that a difference within 4.5 indicates acupuncture is not inferior to Climen® treatment. Using a non-inferiority design, and assuming a power of 90%, an alpha value of 5%, and a population standard deviation of 10, a total of 206 patients will be needed in this study. Each group needs 103 patients (assuming a 20% dropout rate).

#### Inclusion criteria

According to the 2011 criteria of the International Menopause Society [20] and the 2001 criteria of the North American Menopause Society [21], women aged between 40 and 55 who meet the following conditions (Table 2) will be included in this study: menstrual irregularity in the past 12 months, or menstrual period off at least twice in the past 12 months, or amenorrhea 2 to 12 months; suffering any of the following symptoms – vasomotor symptoms (hot flashes, sweats), psychological symptoms (insomnia, migraine, irritability), or genitourinary symptoms (vaginal dryness, dyspareunia); serum follicle-stimulating hormone levels >10 units/L, or decline of estradiol after amenorrhea; and voluntarily participating in this study with a written informed consent form signed by themselves.

**Table 2:** Eligibility criteria

**Inclusion criteria**	**Exclusion criteria**
• 40 to 55 years old	• Non-natural menopause (due to surgery, radiation, medication)
• Perimenopausal female (menstrual irregularity in past 12 months; or menstrual period off at least twice in past 12 months; or amenorrhea of 2 to 12 months)	• Use of HRT, SSRIs, vitamin E, or black cohosh in past 4 weeks
• Suffering any following perimenopausal symptoms: vasomotor symptoms (hot flashes, sweats), psychological symptoms (insomnia, migraine, irritability), or genitourinary symptoms (vaginal dryness, dyspareunia)	• Any contraindication of HRT (for example, family history of breast cancer, hysteromyoma, benign galactophore diseases, or ovarian cyst)
• Serum FSH level >10 units/L, or decline of estradiol after amenorrhea	• Unknown causes of vaginal hemorrhage
• Willing to receive treatment and sign consents	• Poorly controlled hypertension, diabetes mellitus
	• History of malignant cancer, mental disorders (including depression), thyroid diseases, diabetic neuropathy
	• Dermatosis (for example, eczema, psoriasis)
	• Serious heart, liver or kidney diseases
	• Coagulation defeats or use of anticoagulant medicine (for example, warfarin, heparin, and so forth)
	• Use of sedative or anti-anxiety drugs
	• Cigarette smoking, alcohol or drug abuse
	• Pacemaker or artificial joints
	• Pregnant women or women in lactation
	• Participation in other clinical trials

FSH, follicle-stimulating hormone; HRT, hormone replacement therapy; SSRI, selective serotonin reuptake inhibitor.

#### Exclusion criteria

Patients with any of the following conditions (Table 2) will be excluded: non-natural menopause, due to surgery (for example, hysterectomy with ovarian conservation, and hysterectomy with bilateral oophorectomy; because the severity or patterns of symptoms may differ from the natural menopause, we therefore exclude these participants [22,23]), radiation, or medication; use of HRT, selective serotonin reuptake inhibitors, vitamin E or black cohosh (which was reported may alleviate menopausal symptoms [24]) in the past 4 weeks; any contraindication of HRT (for example, family history of breast cancer, hysteromyoma, benign galactophore diseases, or ovarian cyst); unknown causes of vaginal hemorrhage; poorly controlled hypertension, diabetes mellitus; history of malignant cancer, mental disorders (including depression), thyroid diseases, diabetic neuropathy; dermatosis (for example, eczema, psoriasis); any serious diseases of the heart, liver, or kidney (there may be risks that acupuncture induces stress action and thus harms patients with serious illness); coagulation defeats or use of anticoagulant medicine (for example, warfarin, heparin, and so forth – the acupuncture procedure involves penetration of the skin, we therefore will exclude participants with bleeding problems); use of sedative or anti-anxiety drugs; cigarette smoking, alcohol or drug abuse; artificial joints or heart pacemaker; pregnant women or women in lactation (it is still unclear whether acupuncture will lead to abortion or other side effects); and participation in other clinical trials.

#### Recruitment procedures

Three strategies will be used to recruit participants with perimenopausal syndrome. The first is to recruit participants in outpatient clinics from the three hospitals. The chief physicians from the departments of gynecology and acupuncture in each hospital were invited to attend a discussion of how to recruit patients. Research assistants will be sent to their departments to help screening participants. Moreover, posters of this trial will be shown outside the clinics to attract possible candidates. Second, we will post advertisements through television broadcasts, newspapers, and so on. In these advertisements, we will briefly introduce the population we want to include, and mention the free treatment for participants who are eligible. Third, we will organize several education groups for women experiencing the perimenopausal period; introducing these people to some basic knowledge and healthcare, as well as this trial in order to attract possible patients who want to participate.

### Interventions and comparison

#### Rationale for acupuncture and auricular acupressure protocol

The acupuncture plus auricular acupressure protocol in this trial was formed in two steps: first, we screen for a range of acupuncture points, auricular points, and stimulation methods through a systematic review of record on ancient books and acupuncture textbooks, as well as results of published articles [25]. Second, meetings were held to further screen for a standardized acupuncture protocol and consensus reached among acupuncture experts in China. Based on the theory of acupuncture and TCM, perimenopausal syndrome is caused by kidney deficiency, exhaustion of *Tiangui* (sex promoters), deficiency of essence and blood, depletion of the Chong and Ren meridians, imbalance between Yin and Yang of the kidney, and dysfunction of the viscera. According to TCM differentiation, perimenopausal syndrome corresponds to the main pattern of kidney Yin deficiency, kidney Yang deficiency, kidney Yin and Yang deficiency, liver and kidney deficiency, disharmony between heart and kidney, and spleen and kidney Yang deficiency [26,27]. We therefore chose Guanyuan (RN4), Zigong (EX-CA1), Zusanli (ST36), and Sanyinjiao (SP6) as the primary acupuncture points. In addition, subsidiary acupoints based on the different symptoms of each patient are Hegu (LI4) and Fuliu (KI7) for hot flashes and sweats; Baihui (DU20) and Anmian for sleep disorders; Taichong (LR3) and Shenmen (HT7) for mood disorders; Fengchi (GB20) and Tinggong (SI19) for dizziness and tinnitus; Ligou (LR5) and Taixi (KI3) for vaginal dryness and painful intercourse; and Xuanzhong (GB39) and Yanglingquan (GB34) for aching bones. All of the acupoints chosen above, except Guanyuan (RN4) and Baihui (DU20), which are located in the middle of line, are needled bilaterally. According to the theory of TCM, we also choose auricular points as follows: heart (CO_15_), liver (CO_12_), kidney (C0_10_), spleen (CO_13_), endocrine (CO_18_), internal genital (TF_2_), subcortex (AT_4_), Er Shenmen (TF_4_), sympathetic (AH_6a_), and apex of antitragus (AT_1, 2, 4i_) (Table 3).

**Table 3:** Acupuncture points and auricular points

**Point**	**Primary**	**Additional**
Acupuncture	Guanyuan (RN4)	Hegu (LI4), Fuliu (KI7)
	Zigong (EX-CA1)	Baihui (DU20), Anmian
	Zusanli (ST36)	Taichong (LR3), Shenmen(HT7)
	Sanyinjiao (SP6)	Fengchi (GB20), Tinggong (SI19)
		Ligou (LR5), Taixi (KI3)
		Xuanzhong (GB39), Yanglingquan (GB34)
Auricular	Heart (CO_15_)	
	Liver (CO_12_)	
	Kidney (C0_10_)	
	Spleen (CO_13_)	
	Endocrine (CO_18_)	
	Internal genital (TF_2_)	
	Subcortex (AT_4_)	
	Er Shenmen (TF_4_)	
	Sympathetic (AH_6a_)	
	Apex of antitragus (AT_1, 2, 4i_)	

### Acupuncture plus auricular acupressure treatment group

All acupoints are punctured by filiform needles when patients are lying in a comfortable supine position. Sterile, disposable Hwato needles (provided by Suzhou Hwato Medical Instruments Co. Ltd, Suzhou, China) are used in this study; they are 25 to 50 mm in length and 0.30 mm in diameter. The needle is inserted with the double hand-needle insertion technique. The depth of insertion is adjusted based on the standard permissible depth of insertion for each acupoint. The needle is then twirled, rotated, lifted and thrust moderately until achieving De qi sensation. The needles are retained for 30 minutes in every session and manipulated twice every 10 minutes with intermittent stimulation. Each manipulation lasts for 10 seconds. All needles are taken out with clean cotton balls to avoid bleeding after 30 minutes. Every patient will receive 28 sessions of acupuncture in total over a period of 12 weeks: three sessions per week in the first 4 weeks, every 2 days with a 1-day interval; and two sessions per week in the following 8 weeks, every 3 days with a 2-day interval.

All of the auricular points will use plaster therapy. One piece of Semen Vaccariae is put in the middle of one piece of 0.5 cm × 0.5 cm medical adhesive plaster. After sterilizing the skin of the auricular points, the plasters are stuck to the points with hemostatic forceps. The patients are required to press the auricular points gently by themselves three to five times a day lasting 1 minute, especially pressing them half an hour before sleep for 2 minutes, until the patients have distention, soreness, numbness, and warm sensation with the local auricular skin congestive, flashed and hot. The strength of pressing should consist of the individual’s endurance. The plaster with Semen Vaccariae is exchanged for a fresh set twice weekly for 12 consecutive weeks, every 3 days with a 2-day interval, both ears used alternately.

### Climen® control group

The patients are prescribed Climen® (complex packing estradiol valerate with cyroterone acetate tablets; Bayer Healthcare Company Limited) for three cycles: a 28-day sequential therapy combing estradiol valerate 2 mg/day (white tablets) from day 1 to day 11, and estradiol valerate 2 mg/day plus cyroterone acetate 1 mg/day (pink tablets) from day 12 to day 21, followed by a 7-day treatment-free interval.

### Outcome measurement

The primary outcome of this trial is the MRS, which is widely used for evaluating the severity of aging symptoms. Eleven questions of the MRS can be classified into three categories: psychological, somatic, and urogenital. A higher score indicates worse symptoms. The primary outcome is measured at randomization and another five points (4, 12, 16, 24, and 36 weeks after randomization). The secondary outcomes include the following items: Menopause-Specific Quality of Life; average hot-flash score during 24 hours; and the level of estradiol, follicle-stimulating hormone, and luteinizing hormone in serum. The first two secondary outcomes are measured at randomization and 4, 12, 16, 24, and 36 weeks after randomization. The other secondary outcomes are measured before randomization and 12 and 24 weeks after randomization.

All patients are asked to fill in a 7-day hot flash diary before randomization and 4, 12, 16, 24, and 36 weeks after randomization. After treatment, patients will be encouraged to finish these diaries by short messages, telephone calls, and emails in order to avoid selective loss to follow-up.

To exclude patients who have serious heart, liver, and kidney diseases, all participants will also receive routine tests of blood, liver function, kidney function, and electrocardiogram before randomization. Patients will also receive breast ultrasound and gynecological B ultrasound to exclude some uncovered galactophore and gynecological diseases. Meanwhile, aiming to evaluate the side effects of both methods, participates will receive the following items after the end of treatment: breast ultrasound and gynecological B ultrasound; and liver function (alanine aminotransferase, aspartate aminotransferase) and kidney function (blood urea nitrogen, serum creatinine).

This study will also estimate the expectations of participants using the credibility and expectancy questionnaire, aiming to clarify whether expectations make an impact on outcomes.

All patients in this study are required to document whether or not they take other medicine to relieve their perimenopause-related symptoms during 36 weeks, including the name and dosage of the medicine, the time and duration of taking, the reasons and the side effects of the medicine.

During the trial, all adverse events are to be recorded during treatment and the follow-up period, including bleeding, hematoma, fainting, serious pain, local infection, and so on, caused by acupuncture; and irregular vaginal bleeding, breast tenderness, headaches, nausea, and so on, caused by Climen®. Serious adverse events associated with the trial should be reported to the principal investigator immediately. All details will be documented, treatment options for rescue will be given at once, and appropriate action will be taken to prevent recurrence.

All acupuncturists were qualified to perform this trial after they participated in special training classes and passed the examination. The special training classes were held twice in August 2013, aiming to make all of the acupuncturists well understand all details of this trial. For instance, they were trained to use the central randomization method, to fill in the case report form, to find the correct points, to manipulate the needles, and so on. Additionally, to ensure the quality of this trial, the clinical monitors will check the processes of the trial and document the details of the processes once a month in every hospital. Moreover, monitors named by the principle investigator will check the accuracy and validity of the original data from the clinical centers. Regular meetings will be held to consider the recruitment rate, adverse events, protocol violations, difficulties and problems appearing during this study, and so on.

Patient drop-outs, their reasons, and their compliance will be recorded at the 36th week after randomization. All patients will be recorded until the end of this trial.

### Statistical analysis

All data from this study will be analyzed by special analysts with SPSS 20.0 (IBM Corp., Armonk, NY, USA).

The tests of outcomes are based on the intention-to-treat population. Sensitivity analyses are performed for the primary outcomes (MRS measured 16 weeks after randomization) by replacing missing data with multiple imputations and lost observations carried forward using SPSS 20.0. Multiple imputations are calculated using the propensity score method. The MRS is used as a dependent variable in an analysis of covariance, with the groups as random factors, and with baseline MRS and age as covariates, to account for potential baseline differences. Adjusted mean treatment effects are given alongside corresponding 95% confidence intervals and *P* values. The same analysis method will be performed for all secondary outcomes.

In addition to the above analysis, we will perform an exploratory analysis to describe group means over time. We will calculate summary statistics to reveal whether the two groups are changing in a similar or different fashion. A two-sided test is applied for available data, and *P* < 0.05 is considered statistically significant.

There is one acupuncturist in each center to do all treatment, which probably introduces a clustering effect in this trial. We therefore calculated the intracluster correlation coefficient from the result of this trial and report the coefficient [28].

## Discussions

This trial is sponsored and financially supported by the National Key Technology R&D Program during the Twelfth Five-year Plan Period of China. The study reported in this article is only one part of this program, which is an international multicenter systematic evaluation of clinical effectiveness of acupuncture. The purposes of this international collaborative program are to prove the therapeutic effects and safety of acupuncture and to establish guidelines for clinical practice. The result of this trial is expected to provide convincing evidence that acupuncture is effective for patients with perimenopausal syndrome.

Previous RCTs show acupuncture in the treatment of symptoms of both perimenopausal and postmenopausal females has no advantage, compared with placebo needles [16-19]. Compared with no medical treatment (for example, self-caring, waiting), however, acupuncture is able to relieve symptoms quite well [16,17,29]. In view of controversial results, some researchers asserted that the placebo needles might have similar effects to verum acupuncture [30], and this view may underestimate the total effectiveness of verum acupuncture. We are therefore conducting a pragmatic trial that requires a positive drug as the control group to clarify an acupuncture therapeutic effect rather than a placebo needle. Moreover, we use a non-inferiority method to estimate the sample size, and only include perimenopausal patients. What we expect is to prove the therapeutic effects of acupuncture on perimenopausal syndrome, and provide another alternative treatment for perimenopausal women.

HRT has been in use for more than half a century and is regarded as a gold standard treatment for vasomotor, urogenital symptoms and their potential consequences such as sleep problems, and mood disorders [31-33]. HRT can improve the health-related quality of life through the alleviation of these symptoms. Meanwhile, HRT is beneficial to prevent aging women from osteoporosis, metabolic syndrome, and cardiovascular problems [4]. HRT is thus an ideal treatment for females with perimenopausal syndrome. Given all patients in this trial have an intact uterus, we choose estrogen plus progestin therapy to negate the risk of endometrial cancer rather than using estrogen alone, and 12-week treatment for women aged 40 to 55 should be safe according to the statement of the North American Menopause Society [4]. Furthermore, studies have shown that sequential estradiol valerate and cyroterone acetate combinations: relieve climacteric complaints via increasing serum estrogen, and provide a good menstrual cycle inducing a physiological secretory transformation of the endometrium without side effects [34]; exert a positive effect on body fat mass and distribution [35]; and may have protective effects for postmenopausal bone loss [36]. We therefore chose Climen® as a positive drug control.

We chose the MRS as the primary outcome, which was initially developed to measure the severity of aging symptoms and their impact on health-related quality of life in the 1990s [37]. Three different dimensions of symptoms (psychological, somato-vegetative, and urogenital symptoms) are measured by 11 items, which can be easily completed by women themselves rather than physicians [37,38]. The total scores of MRS vary between 0 (no symptom) and 44 (highest degree of symptoms) based on the severity of symptoms; the higher scores patients obtain, the more serious symptoms they have. Since 1992 the MRS has been developed and proven to be reliable and valid for decades [39,40]. This self-assessment scale has the following objectives: to enable comparisons of the symptoms of aging between groups of women under different conditions; to compare severity of symptoms over time; and to measure changes before and after treatment [36-38]. Since the English version was published [41], 24 different languages versions are now available on the website [42]. Due to its reliability and validity, we chose the MRS as the primary outcome of this trial to assess perimenopausal syndrome. We chose Menopause-Specific Quality of Life as one of the secondary outcomes to assess the severity of these symptoms affecting patients’ life quality. To measure the severity and frequency of hot flashes and sweats, participates will be required to fill hot-flash dairies for 1 week before each measurement point. The average hot-flash score during 24 hours is the 7-day total score of severity (0, none; 1, mild; 2, moderate; 3, severe)/7.

One recent RCT found that traditional acupuncture continued relieving the vasomotor symptoms throughout the 3-month treatment period, while the sham acupuncture was tending towards stability after the first month [17]. A longer duration of treatment was hypothesized to be a superior effect between traditional and sham acupuncture. Accordingly, in order to observe a significant effectiveness of acupuncture, participants in our study will receive 3 months of treatment. Another study indicated that the effectiveness of acupuncture on perimenopausal syndrome was superior to HRT during the 6-month follow-up period [43]. Therefore, it is necessary to conduct a longer period of follow-up to obverse the continuous effects of acupuncture compared with positive drug, and also to assess the safety after treatment. Before this trial we conducted a questionnaire survey of 156 women with perimenopausal syndrome in the First Affiliated Hospital of Chengdu University of TCM to obtain the public’s acceptance and understanding of acupuncture treatment for perimenopausal problems (the detail of this survey will be available soon). According to the results of the survey, previous RCTs and the opinions of experts, we designed this protocol for better treatment and follow-up arrangements.

Our study still has limitations. Firstly, the whole research period of this study is 37 weeks (1 week baseline, 12 weeks of treatment and 24 weeks of follow-up) and may increase the rate of patient drop-out. We therefore reduce the treatment demand after the first month of treatment and keep close contact with our patients via telephone calls, short messages, emails, and so forth, during the follow-up period, to decrease the drop-out rate. Secondly, the three centers of this study are in Chengdu, which is a capital city located in the southwest of China, and the patients we will include in this trial are mostly local residents. The population may therefore not represent the advantages of a multicenter study. Thirdly, participants assigned to the acupuncture group may have more opportunities for contact with their acupuncturists and build a good doctor–patient relationship rather than those of the control group, which may improve the therapeutic effect [44]. We will therefore keep close contact with patients in the control group by telephone, Internet, and so on, to decrease this potential bias. Meanwhile, it is difficult to blind patients after randomization due to the specificity of acupuncture. We therefore strictly conduct central randomization and appoint different people to perform therapy, measurement and statistical analysis.

Owing to a lack of solid evidence for effectiveness of acupuncture treatment on perimenopausal syndrome, this trial aims to clarify this issue by means of a multicenter randomized controlled large-scale trial.

In conclusion, the results of this trial are expected to confirm whether acupuncture is effective in relieving perimenopausal syndrome.

## Trial status

This trial is currently in the recruitment phase.

## Abbreviations

HRT: hormone replacement therapy; MRS: Menopause Rating Scale; RCT: randomized controlled trial; TCM: traditional Chinese medicine.

## Competing interests

The authors declare that they have no competing interests.

## Authors’ contributions

YL, HZ, LZ, QZ, ZSL and BYL participated in the conception and design of this trial, and in plans for the data. YL, HZ and QHZ were responsible for planning to draft and revise the manuscript. LZ and ZSL are monitors of this study. QHZ, EQQ, YW, QZ, HBZ, YZ, WS and XXZ are responsible for the recruitment and treatment of patients in each center. EQQ collects the data. All authors read this manuscript and approved the publication of this protocol.
